# Establishing preclinical models for clear cell sarcoma of soft tissue

**DOI:** 10.3389/fonc.2025.1589773

**Published:** 2025-06-18

**Authors:** Bingbing X. Li, Jake Piesner

**Affiliations:** ^1^ Program in Chemical Biology, Department of Chemical Physiology and Biochemistry, Oregon Health and Science University, Portland, OR, United States; ^2^ Knight Cancer Institute, Oregon Health and Science University, Portland, OR, United States

**Keywords:** clear cell sarcoma of soft tissue, CCSST, *EWSR1*, *ATF1*, *CREB* metastasis, invasion

## Abstract

**Introduction:**

Clear cell sarcoma of soft tissue (CCSST) is a rare but aggressive soft tissue sarcoma driven by fusion proteins. The translocation of t(12;22) or t(2;22) leads to fusion formation between Ewing Sarcoma Breakpoint Region 1 (*EWSR1*) and either activating transcription factor 1 (*ATF1*) or cAMP-response element-binding protein (*CREB*). Several fusion types have been discovered in CCSST patients. Only type 1 EWSR1-ATF1 fusion is known to be a constitutively active transcription factor. However, the transcriptional activity of other fusion types remains unknown. In addition, there is a significant lack of preclinical *in vivo* metastasis models for CCSST.

**Methods:**

We evaluated the transcriptional activity of seven EWSR1-ATF1 and one EWSR1-CREB fusion proteins using reporter assays. Migration and invasion assays were performed in CCSST cell lines. To model metastasis *in vivo*, CCS292 cells expressing firefly luciferase were injected intravenously into NSG mice, and metastatic spread was monitored weekly by bioluminescence imaging.

**Results:**

We show that type 1, 2, 3 and 7 EWSR1-ATF1 fusions, as well as EWSR1-CREB are constitutively active while type 4, 5 and 6 are not. Among the four CCSST cell lines tested, only CCS292 showed invasion and migration potential, despite all lines harboring EWSR1-ATF1 fusions. CCS292 cells with firefly luciferase expression developed robust metastasis *in vivo*.

**Conclusion:**

All the in-frame fusions are constitutively active. We developed both *in vitro* and *in vivo* models of CCSST metastasis based on the CCS292 cell line, which are valuable tools for assessing potential therapeutics for CCSST patients.

## Introduction

Clear cell sarcoma of soft tissue (CCSST), first described in 1965 (1), is a rare type of soft tissue sarcoma primarily affecting adolescents and young adults. It typically arises in lower extremities. CCSST has clinical and histological similarities to malignant melanoma (2). However, these two cancers are two different disease entities having different prognosis and responses to therapeutics (3, 4). Both CCSST and malignant melanoma arise from neural crest cells and express melanocytic markers such as S100, human melanoma black 45 (HMB45), melan A. The definitive diagnosis depends on the distinct genetic hallmark of CCSST, which harbors a balanced t(12;22) (q13;q12) translocation (5, 6). This chromosome translocation leads to fusion between Ewing sarcoma breakpoint region 1 (*EWSR1*) and activating transcription factor-1 (*ATF1*) to create an oncogene *EWSR1-ATF1* (5).

ATF1 is a member of the cAMP-responsive element (CRE) binding protein (CREB) family transcription factor. This family of transcription factors have a basic region and leucine zipper (bZIP) domain at the C-terminus to bind DNA (7, 8). Transcription activation of this family of factors requires phosphorylation at Ser133 of CREB or Ser63 of ATF1 by protein Ser/Thr kinases including cAMP-regulated protein kinase A (PKA) (9). However, the N-terminal EWSR1 domain contains a transcription activation domain independent of cAMP (10–12). We and others have demonstrated that the fusion protein EWSR1-ATF1 is constitutively active to drive the expression of target genes that are normally regulated by CREB/ATF1 (13–16).

The estimated 5-year overall survival for CCSST is around 50% and the 5-year survival rate is only ~20% for metastatic disease (17–20). There are no approved therapies for CCSST, which is notoriously known for its insensitivity to current chemotherapies and immunotherapies (17, 21–25). Targeted therapy crizotinib was clinically evaluated in Phase II trial for CCSST. However, it did not demonstrate significant clinical benefits (26). Currently, surgical resection remains the only treatment option for patients. Given the absence of standard of care for this disease, there is a critical need to identify novel therapies for CCSST. In order to achieve this goal, it will be necessary to have appropriate assays for assessing the preclinical activities of putative therapies. It has been shown that EWSR1-ATF1 is necessary and sufficient to induce tumor formation resembling human CCSST pathologies (27, 28). Therefore, specific and effective therapies for CCSST should potently inhibit EWSR1-ATF1’s activity. CCSST shows a strong tendency for local recurrence and distant metastasis and once metastasized, the prognosis is much worse. Therefore, effective therapies are also anticipated to be able to manage metastasis. Being a rare disease, many of the necessary preclinical assays have not been developed. In this report, we present a suite of preclinical assays and models that can be deployed to develop therapies for CCSST.

## Results

### *In vitro* assays to evaluate the transcription activity of fusion genes in CCSST

**Figure 1:**
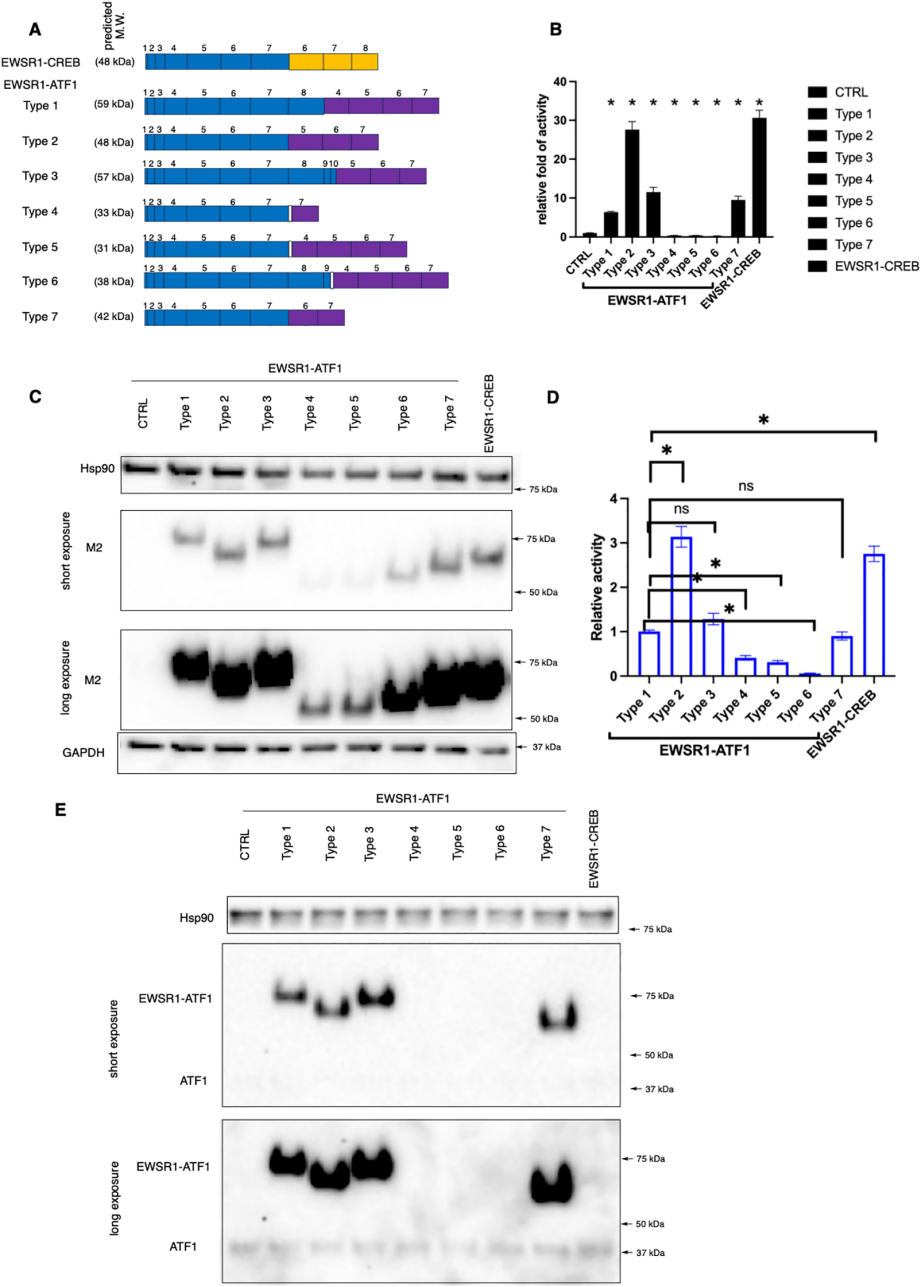
Transcription activity of different EWSR1-ATF1/CREB fusions. **(A)** A schematic summary of known *EWSR1-CREB* and *EWSR1-ATF1* fusion types detected from CCSST patients. Exon numbers for *EWSR1* and *ATF1* or *CREB* are indicated. Among *EWSR1-ATF1* fusions, type 1, 2, 3 and 7 are in-frame fusions while type 4, 5 and 6 are out-of-frame fusions as indicated by a white box between *EWSR1* and *ATF1* exons. *EWSR1* exons are in blue. *CREB* exons are in orange while *ATF1* exons are in purple. The predicted molecular weight of each fusion is also shown. The full amino acid sequence of each fusion type is shown in **Supplementary Figure S1**. M.W., molecular weight. **(B)** Different types of EWSR1-ATF1/CREB fusion displayed different level of transcription activity. HEK293T cells were transfected with indicated fusions along with a CRE-RLuc reporter. Then the RLuc activity was measured and normalized to protein concentration. The RLuc activity of CRE-RLuc-only transfected cells was defined as 1. *P < 0.05 by student *t*-test. Four biological replicates were used to measure luciferase activity, and the experiment was repeated three times. Data are presented as mean ± SEM. Error bars represent SEM. **(C)** HEK293T cells were transfected with indicated Flag-tagged different fusion types. Then the lysates were prepared for western blot. The membrane was incubated with anti-Flag (M2). Anti-Hsp90 and anti-GAPDH were used as loading controls by re-probing the same membrane. **(D)** The relative transcription activity of different EWSR1-ATF1 and EWSR1-CREB fusions. The luciferase activity in B for each fusion was normalized to the protein expression level in **(C)** For comparison purpose, the activity of type 1 was defined as 1.0. Data are presented as mean ± SEM. *P<0.05. Statistical non-significance is indicated as “ns”. **(E)** The lysates from **(C)** were used for western blot with anti-ATF1. Anti-Hsp90 was used as a loading control by re-probing the same membrane. The uncropped blot images are provided in **Supplementary Figure S4**.

The initially discovered *EWSR1-ATF1* is type 1 fusion, where exons 1–8 of *EWSR1* are joined together with the exons 4–7 of *ATF1* (5). Since this initial discovery, six other *EWSR1-ATF1* fusion types and *EWSR1-CREB* fusion have been identified from CCSST patients (**Figure 1A**) (29–31) (30, 32–34). Most CCSST patients carry type 1 *EWSR1-ATF1* fusion (*EWSR1* exon 8/*ATF1* exon 4). The other fusion types are type 2 (*EWSR1* exon 7/*ATF1* exon 5), type 3 (*EWSR1* exon 10/*ATF1* exon 5), type 4 (*EWSR1* exon 7/*ATF1* exon 7), type 5 (*EWSR1* exon 7/*ATF1* exon 4), type 6 (*EWSR1* exon 9/*ATF1* exon 4), and type 7 (*EWSR1* exon 7/*ATF1* exon 6). The predicted protein sequences of the 7 different fusions are shown in **Supplementary Figure S1**. Types 1, 2, 3 and 7 are in-frame fusions with the bZIP domain intact. On the other hand, types 4, 5 and 6 are out-of-frame fusions (**Supplementary Figure S1**). Besides *EWSR1-ATF1* fusions, *EWSR1-CREB* fusion (*EWSR1* exon *7/CREB exon 6*) has also been documented in CCSST cases (35), underscoring the potential importance of CREB/ATF1-driven transcription in the pathogenesis of CCSST.

We and others have reported that type 1 EWSR1-ATF1 fusion is constitutively active in driving CREB/ATF1-mediated transcription activity (13–16). However, the transcription activity of other EWSR1-ATF1 and EWSR1-CREB fusions are unknown. In order to investigate the transcription activity of the EWSR1-ATF1 and EWSR1-CREB, we employed our previously reported transcription reporter assay (16). In this assay, HEK 293T cells were transfected with plasmids expressing a fusion type of interest with a Flag tag and ATF1/CREB transcription reporter plasmid CRE-RLuc, which encodes renilla luciferase (RLuc) under the control of 3 tandem copies of CRE sequence (16). As shown in **Figure 1C**, all the fusions were expressed and migrated at a slightly higher molecular weight (MW) than predicted. Our observed molecular weight shift is consistent with a previous report in the literature (36). It is well-established that wild-type EWSR1, ATF1 and CREB1 migrate more slowly than expected on SDS-PAGE gels. The predicted MW of EWSR1 is 68 kD while the observed MW is ~90 kD (37). The predicted MW of ATF1 and CREB1 is 29 and 35 kD, while the observed MW is ~37 and 43 kD, respectively (16, 38, 39). As a result, the fusions between EWSR1 and ATF1 or CREB also migrated more slowly on the SDS-APGE gels. These differences in migration on SDS-PAGE are possibly due to post-translational modifications (e.g. phosphorylation). The transcription activity of each fusion was determined by the RLuc activity. Consistent with our previous report (16), expression of type 1 resulted in strong constitutive activity (**Figure 1B**). Expression of other in-frame fusion type 2, 3 and 7 also supported potent transcription activation. On the other hand, the out-of-frame fusion type 4, 5 and 6 did not show constitutive transcription activity (**Figure 1B**). Instead, a small but statistically significant reduction of the basal CREB/ATF1 transcription activity was observed. We found that type 4, 5 and 6 were expressed at lower levels when comparing to the other fusion proteins. Therefore, we normalized the observed luciferase activity to the expression level of each fusion. As shown in **Figure 1D**, type 4, 5, and 6 indeed showed substantially lower activity. The EWSR1-CREB fusion also retained the intact bZIP domain and expression of this fusion also resulted in constitutive activity (**Figures 1B, D**). While the expression level of different fusions in the heterologous system varied, this variation was not correlated to the transcription activity. For example, type 2 and type 3 fusions were expressed at the similar level. However, they displayed different level of transcription activity with type 2 showing stronger transcription activation (**Figures 1B–D**). The in-frame EWSR1-ATF1 fusions (type 1, 2, 3 and 7) were also detected by anti-ATF1 recognizing the C-terminal portion of ATF-1 (**Figure 1E**).

The lack of constitutive activity for fusion types 4–6 would argue against their roles in the development of CCSST as other fusions are all constitutively active. However, close examination revealed that all the patients who presented types 4–6 also carried other constitutively active types (30, 32–34). For example, the metastatic lesions from one patient presented 4 different types of fusions (type 1, 2, 5, 6) while only type 1 fusion was present in the primary tumor (33). Our results, together with previous mouse modeling studies (27, 28), support the critical importance of constitutive CREB/ATF1-mediated gene transcription in the pathogenesis of CCSST.

### *In vitro* assays to evaluate migration and invasion potential of CCSST cell lines

Metastasis is a key factor associated with prognosis of CCSST patients. The 5-year survival rate dropped to 25% for stage IV disease (18). Therefore, it is imperative that preclinical models of metastasis is included for CCSST drug discovery effort to identify drugs that are effective in managing cases with metastasis. While mouse genetics modeling studies with EWSR1-ATF1 fusion (type 1) have convincingly demonstrated that the fusion is the driver for the development of CCSST tumor (27, 28), none of the genetic mouse models presented robust metastasis. In order to develop models for CCSST metastasis, we first investigated the migration and invasion potential of patient-derived CCSST cell lines using the transwell Boyden chamber assay (40).

**Figure 2:**
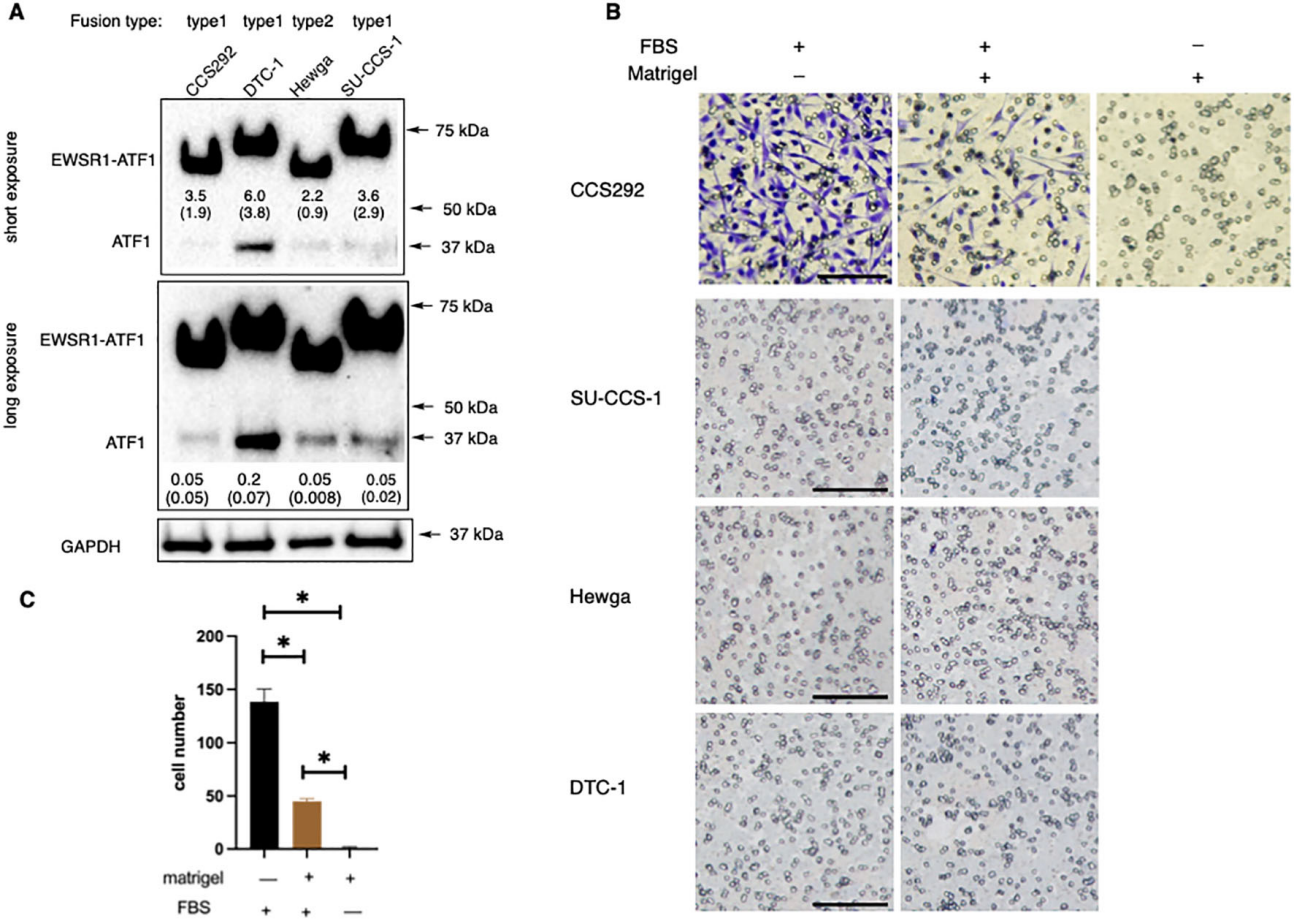
*In vitro* migration and invasion potential of CCSST patient-derived cell lines. **(A)** All the 4 CCSST cell lines express EWSR1-ATF1. The cell lysates were prepared from each cell line and separated on SDS-PAGE for western blotting with anti-ATF1. The top panel is from a short exposure while the middle panel is from a long exposure. Anti-GAPDH (bottom panel) was used as a loading control by re-probing the same membrane. The relative expression levels of EWSR1-ATF1 and ATF1 were quantified by comparing their densitometry values to that of GAPDH. The results of this comparison are labeled beneath the corresponding bands in the short (for EWSR1-ATF1) or long (for ATF1) exposure image. The fusion type present in each cell line is indicated at the top of each lane. The uncropped blot images are provided in **Supplementary Figure S4**. The experiments were repeated twice. **(B)** Migration and invasion of CCS292 cells, SU-CCS-1, Hewga-CCS and DTC-1 cells. The transwell insert was coated with or without Matrigel. The bottom chamber was filled with serum free media or media with 10% FBS. Then CCS292 (3x10^5^/well) cells were seeded in the top chamber after 4 h of FBS starvation and incubated for 24 h The media and cells from the top chamber were gently removed and the membrane was fixed and stained with crystal violet. Representative images are shown under different conditions. Scale bar is 100 μm. **(C)** Quantification of the CCS292 migration and invasion from the images shown in **(B)** data Images of the invaded cells on the membrane were acquired using identical imaging settings to ensure consistency and comparable image sizes. Subsequently, the number of cells that had migrated and invaded through the membrane was quantified by manually counting the stained cells in each image. *P < 0.05 by student *t*-test. Data are presented as mean ± SEM. Error bars represent SEM of 3 different imaging areas. Three independent experiments were performed.

As a rare cancer with limited resources, not many patient-derived CCSST cell lines have been established. We acquired 4 different CCSST patient-derived cell lines: SU-CCS-1, DTC1, CCS292 and Hewga-CCS (**Table 1**). The presence of EWSR1-ATF1 fusion in these cell lines was investigated by western blot of the cell lysates using an ATF1 antibody targeting the C-terminus. As shown in **Figure 2A**, all 4 cell lines harbor EWSR1-ATF1 fusions. Endogenous wild type ATF1 was also present in all 4 cell lines. CCS292 was reported to harbor type 1 fusion (41), however, this fusion migrated faster than the fusion in SU-CCS-1 and DTC-1. We first investigated the migration capability of the cell lines in the transwell Boyden chamber assay (**Figure 2B**). In this assay, the cells are placed in the upper chamber separated from the bottom chamber by a porous membrane. A chemotactic agent, fetal bovine serum (FBS), was present in the bottom chamber but not in the upper chamber (40). If the cells migrate to the bottom chamber, the migrated cells are detected on the membrane upon staining with crystal violet. As shown in **Figure 2C**, when CCS292 cells were placed into the upper chamber, efficient migration towards the bottom chamber was observed. We also screened the migration capability of the other 3 cell lines: SU-CCS-1, DTC1 and Hewga-CCS. As shown in **Figure 2C**, none of these 3 cells lines were able to robustly migrate towards the bottom chamber. Extending the incubation time or increasing the concentration of FBS in the bottom chamber did not yield migrated cells, although occasional DTC1 cells were detected when the incubation time was extended to 48 h and FBS concentration was increased to 20% (**Supplementary Figure S2**). Although comprehensive screenings were not performed, our results indicated that different CCSST cell lines have different migratory potential even though they all carry EWSR1-ATF1 fusion. Other genetic alterations in the cell lines may play a role in their migration potential.

**Table 1:** Characteristics of CCSST cell lines used in this study.

Cell line	Age/sex	Fusion type	Prior treatment	References
SU-CCS-1	16/F	Type 1	Vincristine, actinomycin, cyclophosphamide, dacarbazine, adriamycin, radiation, methotrexate	Ref (42)
CCS292	unknown	Type 1	unknown	Ref (15, 41)
DTC-1	unknown	Type 1	unknown	Ref (14)
Hewga-CCS	34/F	Type 2	Radiation, doxorubicin, ifosfamide	Ref (43)

We further evaluated the invasion potential of the CCSST cell lines. In this case, the porous membrane was first coated with extracellular matrix Matrigel. In order for the cells to reach the bottom chamber, the cells had to invade through the proteinaceous Matrigel and then migrate to the bottom chamber. As shown in **Figures 2B, C**, when CCS292 cells were placed in the upper chamber with Matrigel coating, the cells were able to invade through the Matrigel and reach the other side of the membrane, confirming the invasive potential. Almost no cells could invade and migrate when chemotactic FBS was omitted from the bottom chamber. Consistent with the lack of migration potential of SU-CCS-1, DTC1 and Hewga-CCS, these cells did not present invasive potential either when the membrane was coated with Matrigel (**Figure 2B**). Titrating down the Matrigel concentration did not result in invasion (not shown). Altogether, these results demonstrated that CCS292 is the only cell line among the four CCSST cell lines tested that showed invasion and migration potential while the other three cell lines did not even though they all harbor EWSR1-ATF1 fusion.

### *In vivo* model to evaluate CCSST metastasis

Among the four CCSST cell lines, CCS292 was the only one that demonstrated invasion and migration potential *in vitro*. We investigated if CCS292 could be used for *in vivo* experimental metastasis to be tracked using non-invasive bioluminescence imaging (BLI). To this end, CCS292 cells were transduced with a 3^rd^-generation lentivirus expressing firefly luciferase (Luc). Upon integration and selection using puromycin, CCS292-Luc cells were established and the luciferase activity was stable even when the cells were cultured in the absence of puromycin for an extended period of time (**Supplementary Figure S3A**). Similar to the original CCS292 cells, CCS292-Luc with luciferase expression also demonstrated potential for *in vitro* invasion and migration in the Boyden chamber assay (**Supplementary Figure S3B**), suggesting that these cells might be appropriate for an experimental *in vivo* CCSST metastasis model.

**Figure 3:**
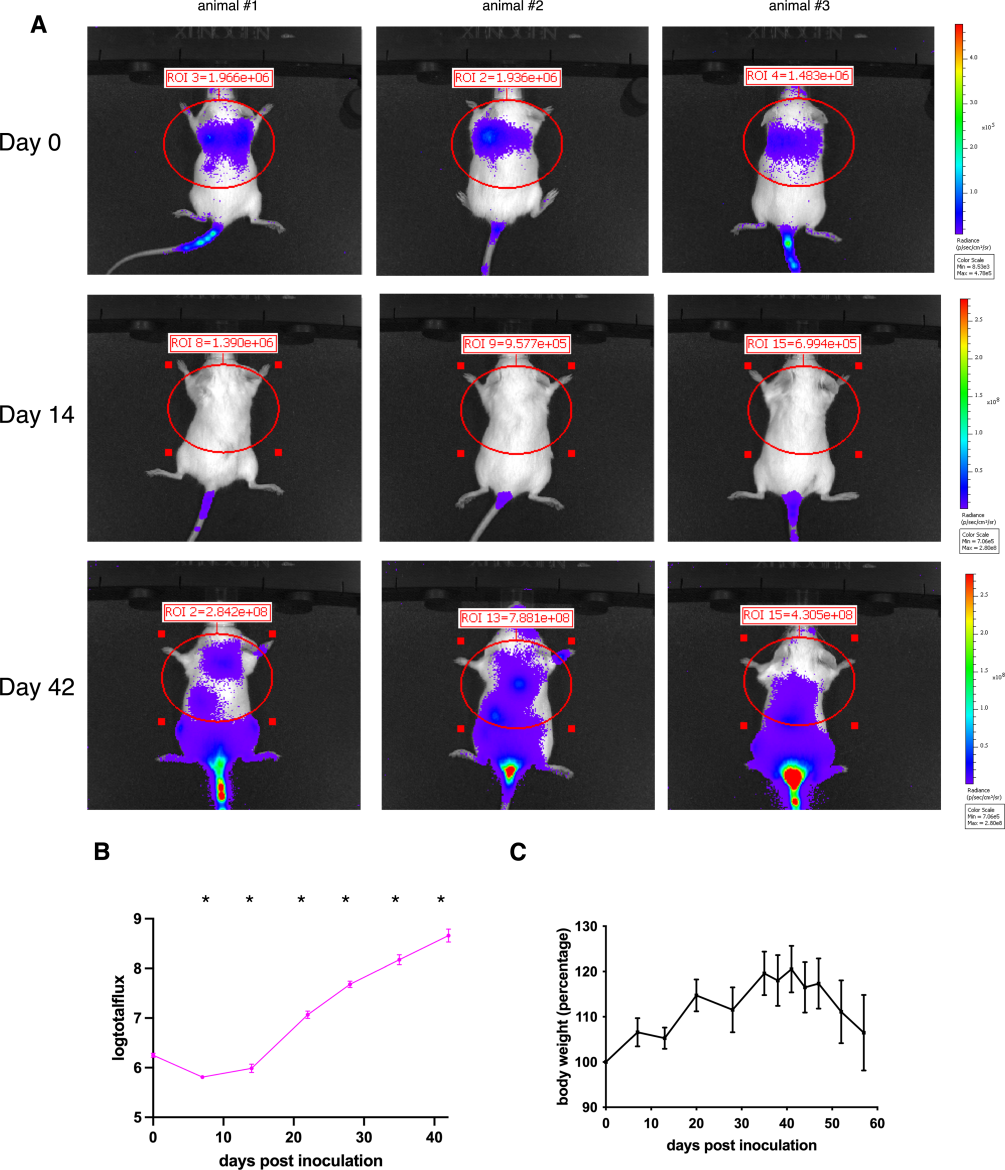
*In vivo* development of metastatic lesions of CCS292-Luc cells. **(A)** Sequential whole-body BLI of NSG mice with CCS292-Luc cells. The cells were injected into three mice through the lateral tail vein. The mice were imaged immediately after injection and weekly thereafter. The Luc activity in the circled region was quantified. Day 0 was defined as the first day of CCS292-Luc cell injection. **(B)** Quantitative summary of bioluminescence signal over time for the NSG recipient mice. The photon flux from the circled region in A was plotted for each mouse over time in log scale. *P < 0.05 by student *t*-test. Data are presented as mean ± SEM. Error bars represent SEM of 3 different mice. This experiment was conducted two independent times. **(C)** The body weight changes of the NSG mice over time. No difference by student *t*-test. Data are presented as mean ± SEM. Error bars represent SEM of 3 different mice. This experiment was conducted two independent times.

To investigate the capability of CCS292-Luc cells to form metastatic lesions *in vivo*, 200,000 CCS292-Luc cells were injected into each immunodeficient NOD *scid* gamma (NSG) mice (n = 3) through the lateral tail vein. Successful injections of the cells through tail vein were verified by BLI immediately after cell injection, when the bioluminescence signals were mainly localized in the lung region (**Figure 3A**). The development of potential metastatic lesions was monitored weekly by BLI. As shown in **Figures 3A, B**, the bioluminescence signal decreased during the initial two weeks, reflecting the loss of majority of the cells in circulation. Starting from week 3, a rapid increase of bioluminescence signal was observed in all 3 mice, suggesting that some cells extravasated into distant organs and started to colonize to form distant metastatic lesions. By week 6, significant bioluminescence signals were detected in multiple areas of the mice. During this period of time, the behaviors of individual mice appeared to be normal, and no abnormal signs were observed. The body weights of the mice during this period were increased slightly (**Figure 3C**). The mice were further monitored for another week. During the final week, they started to exhibit adverse health signs including lethargy, hunched posture, puffed-up furs that required euthanasia. The body weights of the mice also started to decrease slightly (**Figure 3C**).

**Figure 4:**
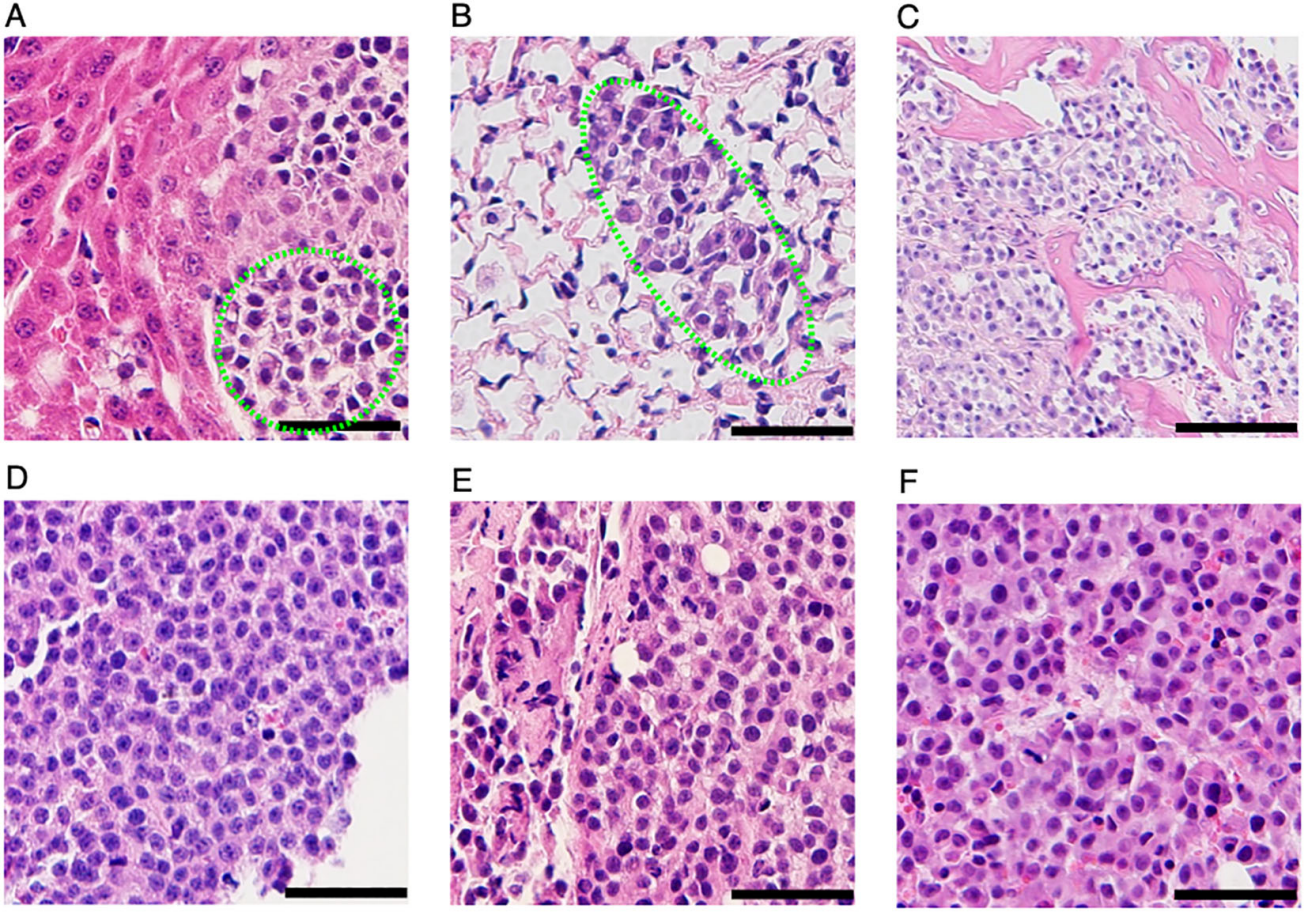
Identification metastatic lesions in NSG mice receiving CCS292-Luc cells with H&E staining. Representative H& E images of liver **(A)**, lung **(B)**, spine **(C)**, visible tumor lesions on the back **(D)**, around the tail **(E)** and knee **(F)**. All the scale bars are 50 μm, except the one in **(C)**, where the scale bar is 100 μm.

Upon euthanasia, gross necroscopy revealed multiple metastatic lesions throughout the body, consistent with the BLI results shown in **Figure 3A**. Visible macrometastases were not observed in the lung. Given the strong bioluminescence signal in the area where lungs are located, the lungs were investigated for the presence of micrometastasis by hematoxylin and eosin (H&E) staining. As shown in **Figure 4B**, cancer cells were present in the lung. All the 3 mice presented micrometastasis in lungs (**Figure 4B**). Two of the 3 mice had micrometastasis in the spine (**Figure 4C**), and one of the mice had liver micrometastasis (**Figure 4A**). Besides the micrometastases, visible tumor nodules were found in areas around the spine in the back (**Figure 4D**), around the tail (**Figure 4E**) and knee (**Figure 4F**). Significant bioluminescence signals were detected in all these regions where tumor nodules were present. Together, these results demonstrated that CCS292-Luc cells were highly metastatic and can be used as an *in vivo* experimental metastasis model for CCSST.

## Discussion and conclusions

CCSST is a rare soft tissue sarcoma without a cure. It is characterized by *EWSR1-ATF1* or *EWSR1-CREB* fusion due to chromosome translocations. While the fusion was discovered more than 3 decades ago, no specific therapies have been developed. To date, seven subtypes of *EWSR1-ATF1* fusion and one type of *EWSR1-CREB* fusion have been discovered from CCSST patients. Among the subtypes, type 1 and type 2 are most commonly detected in patients (2). While it has been demonstrated that type 1 fusion is constitutively active, the transcription activities of the other subtypes have been unknown. Using a CREB/ATF1 transcription reporter assay, we showed that all the in-frame fusions (type 1, 2, 3, 7) and EWSR1-CREB are constitutively active while the out-of-frame fusions (type 4, 5, 6) are not. Even though type 4, 5 and 6 were not expressed well in the heterologous system in HEK 293T cells, their transcription output remained low after normalizing their respective expression level, in comparison to type 1 fusion. The lack of constitutive activity for fusion type 4, 5, 6 suggests that the EWSR1 domain alone does not enhance CREB/ATF1’s transcription activity *in trans* and the intact DNA-binding domain is required to direct the transcription activation domain to the CREB/ATF1-binding sites in the genome. The presence of multiple different fusions is not unique to CCSST. Many alternative forms of EWS-FLI1 exist in Ewing sarcoma (44). Type 1 fusion of EWS-FLI was a significantly weaker transcriptional factor than the other subtypes (45). While initially it was thought that Ewing sarcoma patients carrying type 1 fusion had better prognosis, later larger studies confirmed that the fusion subtyping does not significantly contribute to patient prognosis (46). Due to the small number of CCSST patients, it is unclear if different fusion subtypes correlate to prognosis or metastasis. However, it is interesting to note that all CCSST patients known to carry non-constitutively active fusions (type 4, 5 and 6) also carry one constitutively active fusion, supporting a critical role of constitutive CREB/ATF1-mediated transcription in contributing to the pathogenesis of CCSST.

As a rare disease, the resources for CCSST research are limited. In particular, the preclinical models for CCSST are very limited. While a number of patient-derived cell lines have been reported, the invasion potential of most of the cell lines are unknown. HS-MM cell line was the only one with demonstrated potential for *in vitro* invasion (47). In order to establish scalable preclinical models for CCSST drug discovery, we evaluated the invasion potential of more commonly available CCSST cell lines. Among the four cell lines investigated (SU-CCS-1, DTC-1, CCS292 and Hewga-CCS), only CCS292 demonstrated invasion potential in the Boyden chamber assay, indicating phenotypic heterogeneity among the different CCSST cell lines. Interestingly, both SU-CCS-1 and CCS292 show *c-Myc* copy number gain (41), suggesting that c-Myc is not the driver for CCS292’s metastasis. On the other hand, CCS292 has mitochondrial glutathione transporter *SLCA25A39* copy number gain while SU-CCS-1 does not have (41). Upregulated expression of *SLCA25A39* has been shown to promote colorectal cancer cell migration (48). It remains to be determined if the copy number gain of *SLCA25A39* is critical for CCS292’s migration and invasion capability.

Furthermore, we employed genetically engineered CCS292 cells with luciferase expression to establish an experimental model of CCSST metastasis through tail vein injection. All the mice receiving tail vein injection of the engineered CCS292-Luc cells developed multi-organ metastases that were detectable through BLI and histology analyses. Cancer cell colonization in the lung was observed in all the mice while metastatic lesions were also observed in spine (2 out of 3) and liver (1 out of 3). This resembles the metastatic sites observed in the clinic, where the most common sites of distant metastasis were lung, followed by bone, liver (49). This *in vivo* model of CCSST metastasis is a valuable preclinical model for evaluating experimental therapeutics for CCSST given that distant metastasis is a key prognosis factor for CCSST patients.

Modeling cancer metastasis by directly injecting cancer cells into circulation through tail vein injection is a common method for different types of cancers (50–52). To the best of our knowledge, this modeling has not been investigated in any CCSST cells. It allows rapid evaluation of metastatic colonization and facilitates quantification using assays like BLI. Because it bypasses the requirement to break the original tumor barriers to intravasate, it can facilitate drug testing to quickly create animal cohorts. The major limitation of this experimental metastasis model is that it does not recapitulate the first step of metastasis for cancer cells to intravasate into the blood stream. An alternative to the tail vein injection is the spontaneous metastasis model, where tumor cells are injected orthotopically and then their capability to form distant metastasis is monitored over time. This often requires resection of the primary tumor before metastasis can be detected (53). While this spontaneous model recapitulate all the steps of metastasis including intravasation, extravasation, and recolonization, it does make experiments more complex and time-consuming. Because the animals can develop metastasis at varying rate and the development schedule can be asynchronous, it is more challenging to establish appropriate cohorts for drug testing. It was reported previously that orthotopic tumors derived from HS-MM cells developed metastatic lesions (47). Unlike the model we developed here, the HS-MM model does not allow quantitative non-invasive imaging.

## Experimental section

### Cell lines and culture

HEK293T (54) and SU-CCS-1 (42) cells were purchased from American Tissue Culture Collection (ATCC) and authenticated by STR profiling. DTC-1 (14) was a kind gift from Prof. Torsten Nielsen (University of British Columbia). CCS292 (15) was obtained from Prof. Charles Keller (Children’s Cancer Therapy Development Institute) and Prof. David Fisher (Massachusetts General Hospital). Hewga-CCS (43) was obtained from Prof. Norifumi Naka (Osaka University). DTC-1, CCS292 and Hewga-CCS were not authenticated by STR profiling. The cells were tested for mycoplasma contamination by PCR tests and confirmed to be negative. The cells were routinely maintained in high glucose Dulbecco’s modified Eagle’s medium (DMEM, ThermoFisher) supplemented with 10% Fetal Bovine Serum (FBS, Hyclone) and 10% nonessential amino acids (ThermoFisher) at 37°C with 5% CO_2_. The cells were cultured within 50 passages after thawing.

### Plasmids and antibodies

CREB/ATF1 reporter construct CRE-RLuc was reported previously (55). The fragments corresponding to EWSR1, ATF1 and CREB were amplified from their corresponding cDNA constructs purchased from DNASU plasmid repository (Arizona State University). The backbone of the fusion constructs is pEGFP-N3 (Clontech). Different fragments were assembled together using In-Fusion Snap Assembly Master Mix (Takara). All the final constructs were sequenced verified. Lentiviral luciferase expression vector pLenti CMV Puro Luc was purchase from Addgene. The antibodies used are: anti-Hsp90 (rabbit, Cell signaling Technology, anti-Flag (M2, mouse, Sigma-Aldrich), anti-GAPDH (mouse, Santa Cruz Biotechnology, anti-ATF1 (mouse, Santa Cruz Biotechnology).

### Reporter assay measuring transcription activity

Transfection experiments were performed using Lipofectamine^2000^ (Life Technologies) as previously described (16). Briefly, HEK 293T cells were transfected with CRE-RLuc along with the indicated fusion proteins. Around 24 hours post transfection, the RLuc activity in each transfection was measured using the Renilla Luciferase Assay System (Promega) with FB12 single tube luminometer (Berthold). All the luciferase activity was first normalized to protein concentration followed by further normalization to CRE-RLuc only-transfected cells.

### Migration and invasion assay

Matrigel (Corning) was gently thawed on ice and diluted to 200 μg/mL using cold DMEM. The Transwell inserts (8-μm, Corning) were put in the 24-well plate. For migration assay, the membrane was not coated with Matrigel. For invasion assay, each of the membrane was coated with the diluted Matrigel (120 μL) for about 4 hours at 37 °C. CCS292, DTC-1, Hewga-CCS and SU-CCS-1 cells were harvested and washed with serum free DMEM three times and incubated with serum free DMEM at 37 °C for 4 hr. The cells were collected and 300,000 cells/well or other indicated cell number in serum free DMEM were added to the top chamber. The bottom chamber was filled with 750 μL of DMEM supplemented with 10% FBS. Then the cells were incubated at 37°C for the indicated time period. The media in the top chamber were removed and residual cells were wiped away gently using a wet cotton tip. The inserts were then transferred into a new 24-well plate filled with 1 mL 4% paraformaldehyde for ten minutes at room temperature, which was followed by 1 mL of 70% ethanol for ten minutes at room temperature. The membrane was stained with 0.1% crystal violet for 10 minutes at room temperature followed by washings with 1x PBS (four times). The membrane was gently peeled off to a glass slide. The membrane images were taken on an Olympus microscope using Olympus cellSens software.

### Establishment of CCS292-Luc stable cell line

Lentivirus expressing firefly luciferase was prepared as previously described (16, 56) by co-transfecting HEK 293T cells with pLenti CMV Puro Luc with packaging vectors pMD2.G and pMDLg/pRRE (Addgene) using calcium-phosphate method (Takara). CCS292 cells were transduced with the lentivirus expressing firefly luciferase and selected with puromycin (0.8 μg/mL). The established cells were named as CCS292-Luc.

### Luciferase activity of CCS292-Luc stable cell line

CCS292-Luc cells were cultured under puromycin selection for about two months. An aliquot of the cells was switched to media without puromycin selection for about two months. CCS292-Luc cells with puromycin and without puromycin were harvested. The luciferase activity in each cell was measured using the Luciferase Assay System (Promega) with FB12 single tube luminometer (Berthold). All the luciferase activity was normalized to protein amount.

### Animals

All the animal experiments were approved by Oregon Health & Science University Institutional Animal Care and Use Committee. NSG mice (NOD.Cg*-Prkdc^scid^ Il2rg^tm1Wjl^/*SzJ) were purchased from The Jackson Laboratory. CCS292-Luc (0.2x10^6^ cells/mouse) were injected into three mice through the lateral tail vein under anesthetization. The power analysis to calculate the number of mice was done with G*Power (3.1) (57). We used two-tailed student *t* test with a *P* value <0.05 denoting significance. The following assumptions were made: the standard deviation is 15% of tumor burden (measured by BLI) within the group; the metastatic tumor burden is at least two orders of magnitude above the level on the day of tumor cell injection; the tumor metastasis rate is 100%. A sample size of three tumor-bearing mice will allow us to detect the expected changes with 95% power. The luciferase activity was monitored immediately after injection and weekly thereafter as detailed below. This experiment was independently replicated twice.

### *In vivo* bioluminescence imaging

D-luciferin potassium salt was purchased from GoldBio. A stock solution of D-luciferin was freshly prepared at 15 mg/mL in sterile DPBS (Gibco) and sterilized through a 0.45 um filter. Around 10 minutes before BLI imaging, each mouse received 1.5 mg D-luciferin per 10g body weight intraperitoneally. Images were taken using an IVIS Spectrum (Caliper Life Sciences) with mice under anesthetization. Both dorsal and ventral sides were imaged for each mouse.

### Tissue histology

On day 57 post cell injection, mice were euthanized. The lungs, livers, knee, spines with strong luciferase activity, and visible tumors were harvested. The tissues, except spines and knees, were immediately fixed using 4% paraformaldehyde at room temperature for 24 h, and then sectioned at a thickness of 5 μm. The tissue slides were stained with hematoxylin and eosin (H&E). For the samples with bones, spines and knees, they were immediately fixed using 4% paraformaldehyde at room temperature for 24 h. Then the samples were transferred to 4°C for fixation for another 48 h before undergoing a 3-week incubation in 10% EDTA (pH 7.4) at 4°C for decalcification. The decalcified spine and knee tissues were then sectioned at a thickness of 5 μm and stained with H&E. The slides were imaged on an Olympus microscope using Olympus cellSens software.

### Statistical analysis

The statistical analyses were performed using student *t*-test in Microsoft Excel or Prism 10 (GraphPad). A *P* value of <0.05 was denoted significance.

## Data Availability

The original contributions presented in the study are included in the article/**Supplementary Material**. Further inquiries can be directed to the corresponding author.
