# Supplementary material for: Establishing preclinical models for clear cell sarcoma of soft tissue
Source: Front Oncol. 2025 Jun 18;15:1589773. doi: 10.3389/fonc.2025.1589773 (PMC12213553; doi:10.3389/fonc.2025.1589773)
Supplement: Supplementary file 1 [file DataSheet1.pdf]

EWSR1-CREB amino acid sequence (predicted molecular weight is 48 kDa)

MDYKDHDGDYKDHDIDYKDDDDKASASTDYSTYSQAAAQQGYSAYTAQPTQGYAQT

TQAYGQQSYGTYGQPTDVSYTQAQTTATYGQTAYATSYGQPPTGYTTPTAPQAYSQ

PVQGYGTGAYDTTTATVTTTQASYAAQSAYGTQPAYPAYGQQPAATAPTRPQDGNK

PTETSQPQSSTGGYNQPSLGYGQSNYSYPQVPGSYPMQPVTAPPSYPPTSYSSTQP

TSYDQSSYSQQNTYGQPSSYGQQSSYGQQSSYGQQPPTSYPPQTGSYSQAPSQYS

QQSSSYGQQIAITQGGAIQLANNGTDGVQGLQTLTMTNAAATQPGTTILQYAQTTDGQ

QILVPSNQVVVQAASGDVQTYQIRTAPTSTIAPGVVMASSPALPTQPAEEAARKREVRL

MKNREAARECRRKKKEYVKCLENRVAVLENQNKTLIEELKALKDLYCHKSD

EWSR1-ATF1 type 1 amino acid sequence (predicted molecular weight is 59 kDa)

MDYKDHDGDYKDHDIDYKDDDDKASASTDYSTYSQAAAQQGYSAYTAQPTQGYAQT

TQAYGQQSYGTYGQPTDVSYTQAQTTATYGQTAYATSYGQPPTGYTTPTAPQAYSQ

PVQGYGTGAYDTTTATVTTTQASYAAQSAYGTQPAYPAYGQQPAATAPTRPQDGNK

PTETSQPQSSTGGYNQPSLGYGQSNYSYPQVPGSYPMQPVTAPPSYPPTSYSSTQP

TSYDQSSYSQQNTYGQPSSYGQQSSYGQQSSYGQQPPTSYPPQTGSYSQAPSQYS

QQSSSYGQQSSFRQDHPSSMGVYGQESGGFSGPGENRSMSGPDNRGRGRGGFDR

GGMSRGGRGGGRGGMGKILKDLSSEDTRGRKGDGENSGVSAAVTSMSVPTPIYQTS

SGQYIAIAPNGALQLASPGTDGVQGLQTLTMTNSGSTQQGTTILQYAQTSDGQQILVP

SNQVVVQTASGDMQTYQIRTTPSATSLPQTVVMTSPVTLTSQTTKTDDPQLKREIRLM

KNREAARECRRKKKEYVKCLENRVAVLENQNKTLIEELKTLKDLYSNKSV

EWSR1-ATF1 type 2 amino acid sequence (predicted molecular weight is 48 kDa)

MDYKDHDGDYKDHDIDYKDDDDKASASTDYSTYSQAAAQQGYSAYTAQPTQGYAQT

TQAYGQQSYGTYGQPTDVSYTQAQTTATYGQTAYATSYGQPPTGYTTPTAPQAYSQ

PVQGYGTGAYDTTTATVTTTQASYAAQSAYGTQPAYPAYGQQPAATAPTRPQDGNK

PTETSQPQSSTGGYNQPSLGYGQSNYSYPQVPGSYPMQPVTAPPSYPPTSYSSTQP

TSYDQSSYSQQNTYGQPSSYGQQSSYGQQSSYGQQPPTSYPPQTGSYSQAPSQYS

QQSSSYGQQIAIAPNGALQLASPGTDGVQGLQTLTMTNSGSTQQGTTILQYAQTSDG

QQILVPSNQVVVQTASGDMQTYQIRTTPSATSLPQTVVMTSPVTLTSQTTKTDDPQLK

REIRLMKNREAARECRRKKKEYVKCLENRVAVLENQNKTLIEELKTLKDLYSNKSV

EWSR1-ATF1 type 3 amino acid sequence (predicted molecular weight is 57 kDa)

MDYKDHDGDYKDHDIDYKDDDDKASASTDYSTYSQAAAQQGYSAYTAQPTQGYAQT

TQAYGQQSYGTYGQPTDVSYTQAQTTATYGQTAYATSYGQPPTGYTTPTAPQAYSQ

PVQGYGTGAYDTTTATVTTTQASYAAQSAYGTQPAYPAYGQQPAATAPTRPQDGNK

PTETSQPQSSTGGYNQPSLGYGQSNYSYPQVPGSYPMQPVTAPPSYPPTSYSSTQP

TSYDQSSYSQQNTYGQPSSYGQQSSYGQQSSYGQQPPTSYPPQTGSYSQAPSQYS

QQSSSYGQQSSFRQDHPSSMGVYGQESGGFSGPGENRSMSGPDNRGRGRGGFDR

GGMSRGGRGGGRGGMGAGERGGFNKPGGPMDEGPDLDLVAIAPNGALQLASPGTD

GVQGLQTLTMTNSGSTQQGTTILQYAQTSDGQQILVPSNQVVVQTASGDMQTYQIRT

TPSATSLPQTVVMTSPVTLTSQTTKTDDPQLKREIRLMKNREAARECRRKKKEYVKCL

ENRVAVLENQNKTLIEELKTLKDLYSNKSV

EWSR1-ATF1 type 4 amino acid sequence (predicted molecular weight is 33 kDa)

MDYKDHDGDYKDHDIDYKDDDDKASASTDYSTYSQAAAQQGYSAYTAQPTQGYAQT
TQAYGQQSYGTYGQPTDVSYTQAQTTATYGQTAYATSYGQPPTGYTTPTAPQAYSQ
PVQGYGTGAYDTTTATVTTTQASYAAQSAYGTQPAYPAYGQQPAATAPTRPQDGNK
PTETSQPQSSTGGYNQPSLGYGQSNYSYPQVPGSYPMQPVTAPPSYPPTSYSSTQP
TSYDQSSYSQQNTYGQPSSYGQQSSYGQQSSYGQQPPTSYPPQTGSYSQAPSQYS
QQSSSYGQQKKLLENVAERRKNM

EWSR1-ATF1 type 5 amino acid sequence (predicted molecular weight is 31 kDa)

MDYKDHDGDYKDHDIDYKDDDDKASASTDYSTYSQAAAQQGYSAYTAQPTQGYAQT
TQAYGQQSYGTYGQPTDVSYTQAQTTATYGQTAYATSYGQPPTGYTTPTAPQAYSQ
PVQGYGTGAYDTTTATVTTTQASYAAQSAYGTQPAYPAYGQQPAATAPTRPQDGNK
PTETSQPQSSTGGYNQPSLGYGQSNYSYPQVPGSYPMQPVTAPPSYPPTSYSSTQP
TSYDQSSYSQQNTYGQPSSYGQQSSYGQQSSYGQQPPTSYPPQTGSYSQAPSQYS
QQSSSYGQQKKF

EWSR1-ATF1 type 6 amino acid sequence (predicted molecular weight is 38 kDa)

MDYKDHDGDYKDHDIDYKDDDDKASASTDYSTYSQAAAQQGYSAYTAQPTQGYAQT
TQAYGQQSYGTYGQPTDVSYTQAQTTATYGQTAYATSYGQPPTGYTTPTAPQAYSQ
PVQGYGTGAYDTTTATVTTTQASYAAQSAYGTQPAYPAYGQQPAATAPTRPQDGNK
PTETSQPQSSTGGYNQPSLGYGQSNYSYPQVPGSYPMQPVTAPPSYPPTSYSSTQP
TSYDQSSYSQQNTYGQPSSYGQQSSYGQQSSYGQQPPTSYPPQTGSYSQAPSQYS
QQSSSYGQQSSFRQDHPSSMVYGQESGGFSGPGENRSMSGPDNRGRGRGGFDRG
GMSRGGRGGGRGGMGAGERGGFNKPGEKF

EWSR1-ATF1 type 7 amino acid sequence (predicted molecular weight is 42 kDa)

MDYKDHDGDYKDHDIDYKDDDDKASASTDYSTYSQAAAQQGYSAYTAQPTQGYAQT
TQAYGQQSYGTYGQPTDVSYTQAQTTATYGQTAYATSYGQPPTGYTTPTAPQAYSQ
PVQGYGTGAYDTTTATVTTTQASYAAQSAYGTQPAYPAYGQQPAATAPTRPQDGNK
PTETSQPQSSTGGYNQPSLGYGQSNYSYPQVPGSYPMQPVTAPPSYPPTSYSSTQP
TSYDQSSYSQQNTYGQPSSYGQQSSYGQQSSYGQQPPTSYPPQTGSYSQAPSQYS
QQSSSYGQQTASGDMQTYQIRTTPSATSLPQTVVMTSPVTLTSQTTKTDDPQLKREIR
LMKNREAARECRRKKKEYVKCLENRVAVLENQNKTLIEELKTLKDLYSNKSV

Supplementary Figure 1. The amino acid sequence of different EWSR1-ATF1 and EWSR1-CREB fusions. The N-terminal portion is a Flag tag. The EWSR1 portion is highlighted. ExPASy website was used to calculate the molecular weight of the fusion proteins.

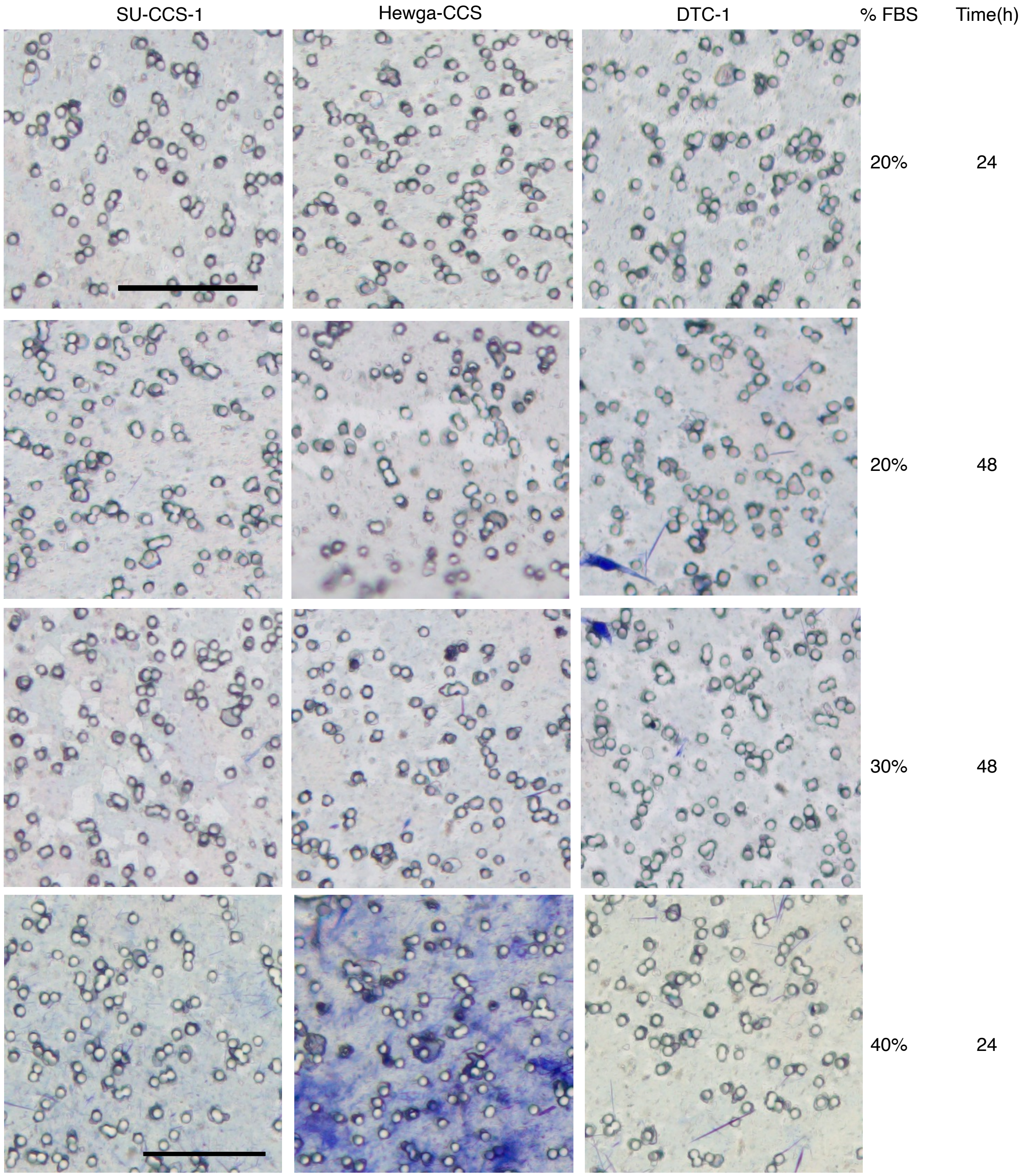

Supplementary Figure 2. Migration of SU-CCS-1, Hewga-CCS and DTC-1 cells. The transwell inserts were not coated with Matrigel. The bottom chamber was filled with

DMEM with indicated concentration of FBS. The cells (3x10^5/well) were seeded in the top chamber after 4 h of FBS starvation and incubated for indicated time periods at 37 ^oC. The media and cells from the top chamber were gently removed and the membrane was fixed and stained with crystal violet. Representative images are shown under different conditions. The scale bars are 100 μm.

A

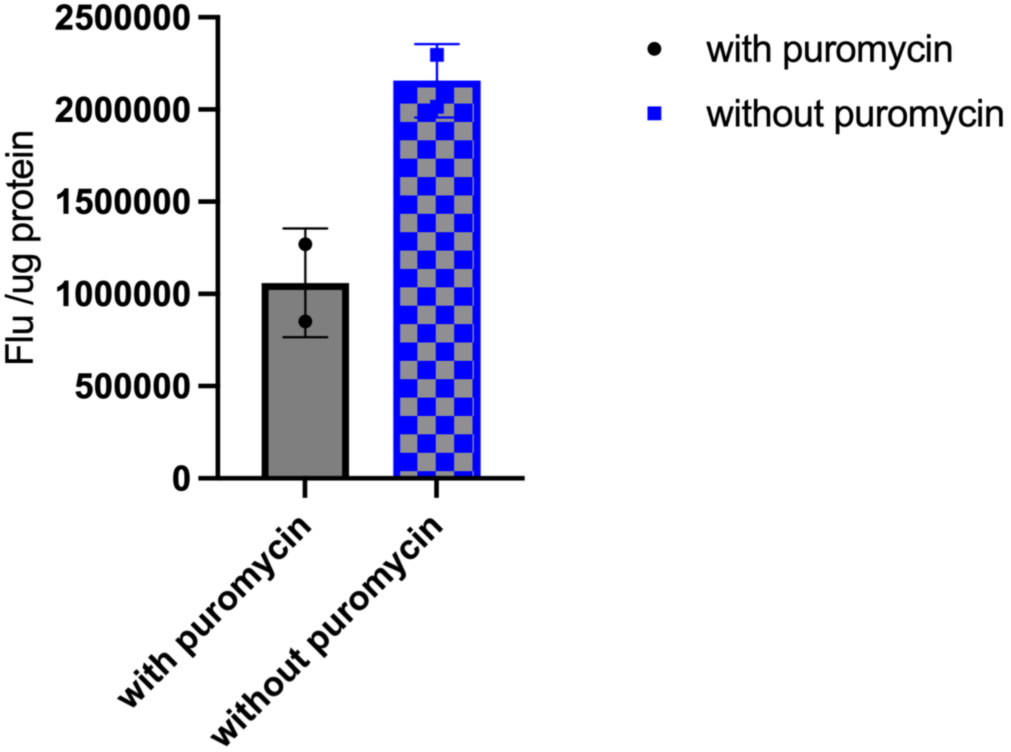
2500000
2000000
1500000
1000000
500000
0
Flu /ug protein
with puromycin
without puromycin
with puromycin
without puromycin

B

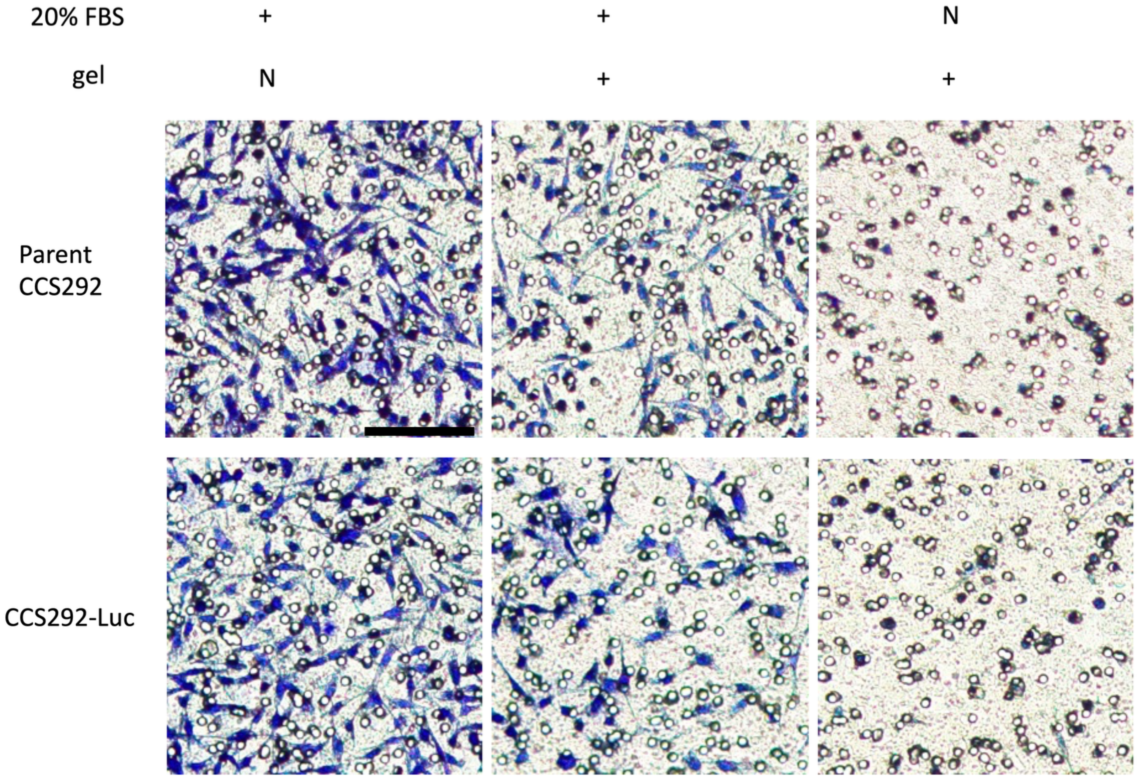
20% FBS
+
+
N
gel
N
+
+
Parent CCS292
CCS292-Luc

Supplemental Figure 3. Establishment of stable CCS292-Luc cells. (A) Firefly luciferase activity of CCS292-Luc cells cultured in the presence or absence of puromycin. (B) Comparison of the invasion and migration ability of CCS292-Luc with parental CCS292 cells. The scale bar is 100 μm.

Figure 1C

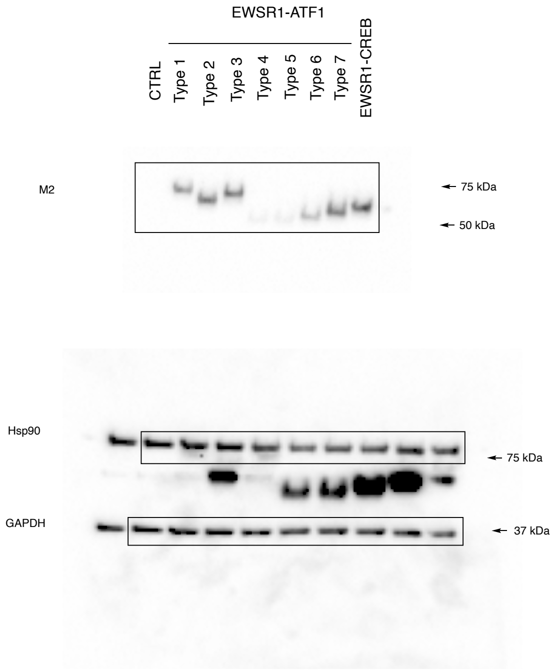

Figure 1D

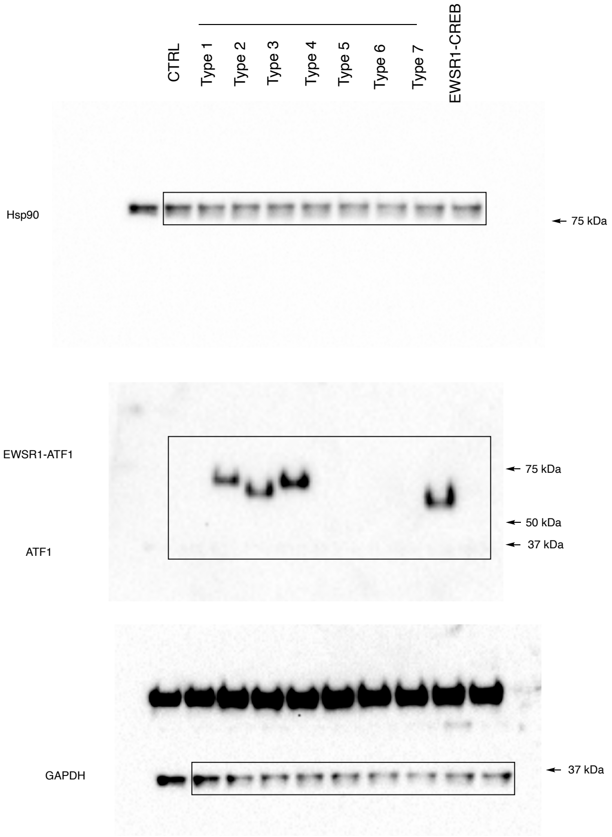

Figure 2A

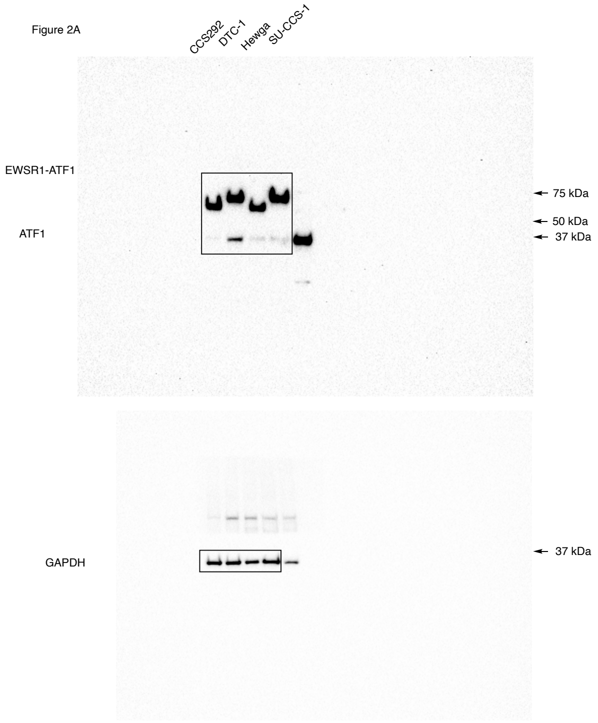

Supplemental Figure 4. Uncropped images of western blot.
